# A Delphi consensus study for teaching “Basic Trauma Management” to third-year medical students

**DOI:** 10.1186/s13049-019-0675-6

**Published:** 2019-10-17

**Authors:** Joana Berger-Estilita, Sabine Nabecker, Robert Greif

**Affiliations:** Department of Anaesthesiology and Pain Therapy, Inselspital, Bern University Hospital, Freiburgstrasse 8-10, 8010 Bern, Switzerland

**Keywords:** Trauma, Teaching, Skills, Undergraduate, Curriculum, Delphi

## Abstract

**Background:**

The Basic-Trauma Management (BTM) course has been taught to third-year medical students in small groups for many years without substantial changes. With the introduction of a new curriculum for Swiss medical students, it was necessary to revise the BTM content and re-align it. Our aim was to identify core competencies for the revised BTM course.

**Methods:**

We applied a three-round step-wise Delphi consensus. First, we asked open-ended questions on what were the most important competencies to be taught for BTM; the second round used Likert scales to ensure agreement on the competencies; and the final round reached out for consensus on these BTM competencies. Stakeholders were selected based on their long-standing experience in teaching BTM and in managing trauma patients.

**Results:**

Consensus was found on 29 competencies out of an initial 130 proposals. “Human Factors”, which had not been taught previously, scored relatively high, at 22%. The sole specific trauma skill agreed upon was the use of tourniquets.

**Conclusions:**

This is an example of curricular revision of a clinical skills course after the introduction of a regulatory framework for undergraduate medical education. The revised course curriculum tailors the concepts and skills in trauma that fulfill stakeholder needs, and are in agreement with the new Swiss learning outcomes.

## Introduction

Competency-based medical education (CBME) is receiving increasing attention worldwide due to societal concerns about the current role of physicians [1–5]. The overarching goal of CBME is to better train and prepare medical students for their medical practice, and to improve patient care [5]. A competency framework has been proposed and guidelines have been developed for undergraduate competency-based medical education [6, 7]. All Swiss medical schools are required to base their undergraduate curricula on a well-defined set of competencies, the so-called “**P**rincipal **R**elevant **O**bjectives and **F**ramework for **I**ntegrated **L**earning and **E**ducation in **S**witzerland” –“PROFILES” [8].. PROFILES is influenced by the CanMEDS 2015 Framework [1] and the Dutch Framework for Undergraduate Medical Education [9], both known frameworks for CBME.

Over the last decade, competency-based curricula have been introduced worldwide in undergraduate medical education. This represents a “shift from the traditional focus on teaching and instruction”, which is also called teacher-centered teaching, to a “learning paradigm that enables students to construct knowledge for themselves”; i.e., student-centered learning [10].

Such changes in educational thinking provide an opportunity to reconsider approaches to undergraduate medical education, although at the same time they can present difficulties as they move beyond routine curricular renewal [11]. These include discussions about the learning of competencies beyond the “Medical Expert” domain. Which outcomes are expected at different stages of student development? Which teaching strategy or method might best achieve the proposed learning outcomes? [3, 11–13] Additionally, educating medical students in complex subspecialties can be challenging, and the optimal timing and content remain unknown [14].

Research is scarce with regard to the process of developing a competency-based undergraduate subspecialty course based on a given framework [15]. Little is known on how the curriculum revision works: who was involved or what pathways were followed to ensure alignment? Although the concept of CBME is not new, its application is still unfamiliar to many medical university faculty members.

The mandatory Basic Trauma Management (BTM) course at the Medical Faculty of the University of Bern in Switzerland, is such an example. This course has been taught for over 15 years to third-year students, exclusively in small groups in a face-to-face 4-h format. It consists of an introductory lesson about trauma management (1 h), and then students are split up into small groups; clinical cases are then discussed, and skills are practiced (3 h). Students receive a “BTM Course-Manual” with facts and skills descriptions for trauma care prior to the start of the course, as their preparation material.

The BTM course was in alignment with the Swiss Catalogue of Learning Objectives for Undergraduate Medical Training (SCLO) [16], which was first issued in 2008. The SCLO focused on knowledge and did not facilitate the acquisition of core practical skills. The 2017, the published Swiss framework, PROFILES [8], defined entrustable professional activities (EPAs), which medical students should perform at the end of their studies. This mandated the revision of the BTM course content. Additionally, unwarranted practice variations had been noted while teaching the BTM course, because tutors devoted different times to lecturing, which resulted in less time for the intended skills training. Finally, these teaching activities at the University of Bern have not been assessed, and concerns have been raised regarding student motivation.

Curriculum revision classically starts with a needs assessment, defined as “the systematic set of procedures undertaken for making decisions about program improvements” [17], which aims to collect data and to narrow the gap between current and recommended practice. On the one hand, the introduction of the Swiss PROFILES represents a legislative need. On the other hand, clinical teachers of BTM expressed needs regarding motivation and the updating of content. Finally, there are normative needs, to diminish unwarranted teaching practice variations. However, the student needs for trauma are unknown. All in all, the educational needs of several stakeholders had to be addressed, and the corresponding competencies appropriate to third-year medical students needed to be explored.

The aim of this study was to find out which competencies are to be taught for the BTM course, and to use the Delphi method to develop a core curriculum for the BTM course at the University of Bern. Our study might be considered as a generalizable example of how to adapt a medical undergraduate curriculum driven by a new regulatory framework.

## Methods

### Study design and setting

We used a three-round modified Delphi technique, with the aim to establish the expected competencies of third-year medical students participating in the BTM course. The Delphi technique allows easy curriculum revision, as investigators can work at a distance with a variety of target group representatives [18, 19], and it provides opinions from a broad range of experts to be consolidated into a manageable number of precise statements. This technique defines that “pooled intelligence” captures the collective opinion of stakeholders [20]. Briefly described, stakeholders answer several rounds of questionnaires, after which an external facilitator provides a summary of the forecasts. In this way, stakeholders can revise their former answers in light of the replies of others, with the chance that the group will converge towards a “consensus” [21].

### Hypothesis and research question

Our a-priori hypothesis was that different stakeholders would have different perspectives on the importance of different topics in BTM. Following this concept, our research question asked “Which trauma topics should be addressed in a basic trauma management course for third-year medical students?”

### Data collection and management

We followed thre Garavalia method for the Delphi technique [22]. In the first round, we asked *open-ended questions* with the scope of prioritizing the most important teaching topics for the BTM course regarding knowledge, skills, and attitudes: “What should be the priorities of the course?” Participating stakeholders were asked to list up to nine “most relevant” items on knowledge (3), skills (3), and attitudes (3) that the third-year medical students should learn in the BTM course. All of the participating stakeholders were invited by e-mail to answer the questionnaire. An e-mail reminder was sent 10 days after the initial invitation.

To ensure a high-quality survey instrument, all of the rounds of questionnaires were developed iteratively by consultation and feedback. The online version was pilot tested with two German-speaking stakeholders (SN, RG) to confirm the comprehensibility of the questionnaire and the usefulness of the response options.

After completion of the first round, the facilitator (JBE) read all of the answers to the open questions, edited, and merged similar answers, and grouped them into categories, to compile the second-round questionnaire.

The round 2 response format was a five-point Likert scale: 1, strongly disagree; 2, moderately disagree; 3, neither agree nor disagree; 4, moderately agree; and 5, strongly agree. This online questionnaire was pilot-tested for ease of completion and technical functioning. At the end of round 2, a final item list was developed. All of the stakeholders who participated in round 1 were invited by e-mail to the second online survey to rate each statement.

After the second round, we calculated the median and interquartile range (IQR) for each statement, as well as the percentage of agreement. by adding together the *4 (Agree)* and *5 (Strongly Agree)* responses, and checking their proportionate part of the total answers for each given question. The predefined cut-off for consensus was 75% agreement and a median score of 5. Items with agreement and consensus were added to the final list. Items with disagreement (median ≤ 3) were excluded.

**Fig. 1:**
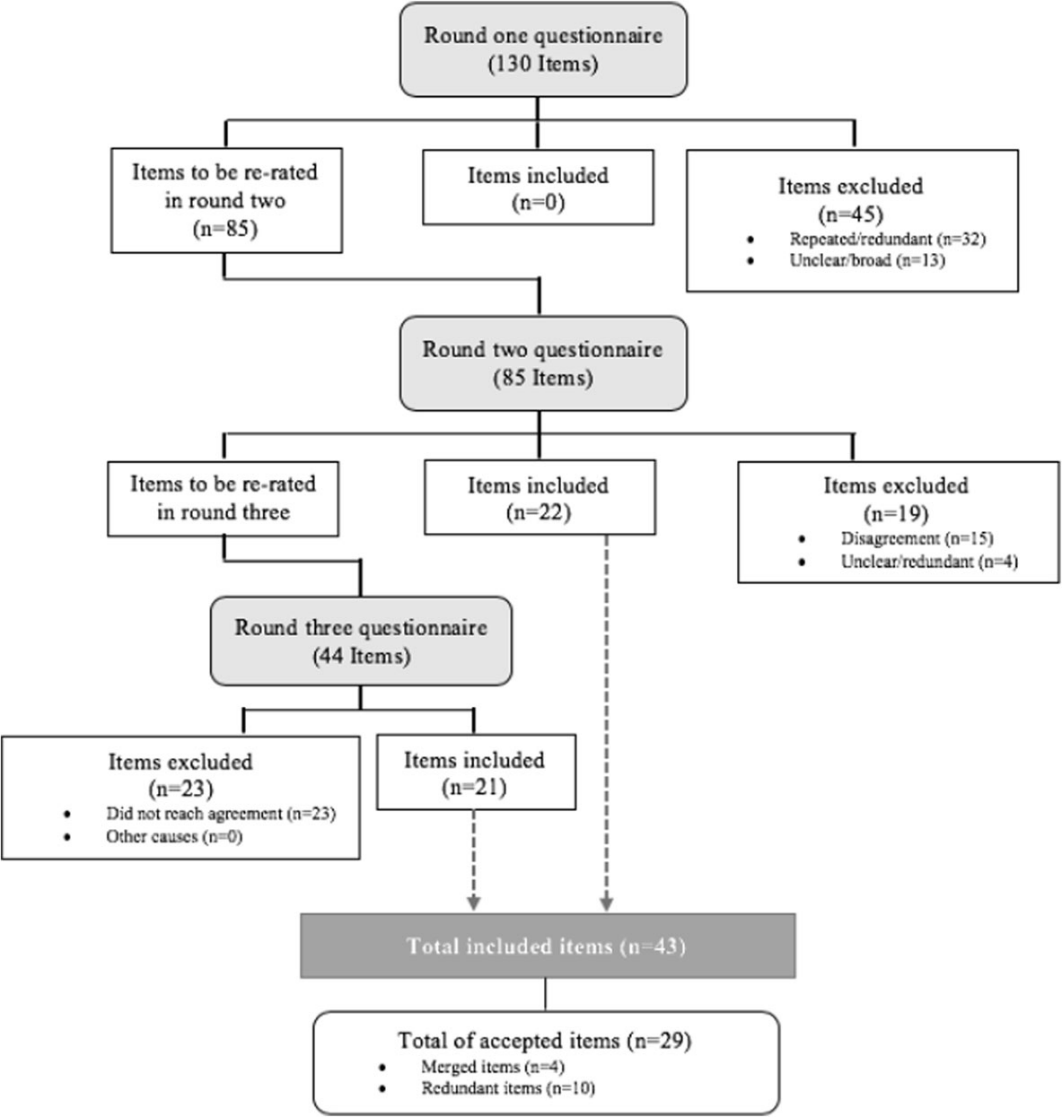
Delphi method flowchart

In round 3, the stakeholders were given the median ratings from round 2 and the levels of agreement for each statement. All items without consensus had to be re-rated. Items included in the questionnaire were again re-piloted, and final edits were made based on the feedback received. In round 3, participants could only answer “yes” or “no”, to decide whether or not the remaining items should be included in the final competence list. Competencies with 75% or more of stakeholder agreement were selected. The pre-final list was sent again to all of the stakeholders to be commented on and signed (Fig. 1).

### Study participants

Stakeholders included a selection of (i) BTM teachers; (ii) certified in-hospital emergency physicians; (iii) final-year medical students who had participated in the BTM course; (iv) out-of-hospital emergency physicians; (v) curriculum designers; and (vi) external educational experts. Selection was based on the long-standing experience of the participants in BTM teaching and their management of trauma patients. We aimed to include 15 stakeholders as participants [20], two to three in each group. A set of 36 invitations to stakeholders was sent out to obtain sufficient participants.

### Gap analysis

We performed gap analysis to compare our findings from the survey to pre-selected, trauma-related objectives and EPAs from the PROFILES [8] report. JBE and RG selected eight general objectives (GO 1.5, GO 1.6, GO 1.11, GO 2.2, GO 3.1, GO 3.2, GO 7.1, GO 7.2) and twenty-two EPAs (EPA 1.1, EPA 1.3, EPA 1.5, EPA 2.1, EPA 2.2, EPA 2.3, EPA 2.4, EPA 2.6, EPA 2.7, EPA 3.2, EPA 5.1, EPA 5.2, EPA 5.3, EPA 5,4, EPA 5.5, EPA 6.1, EPA 6.2, EPA 6.3, EPA 6.5, EPA 6.8, EPA 9.2) that might be covered in the BTM course.

### External review

We used Penciner’s advice (2011) to externally review our results upon completion of the data handling. Three trauma management experts were selected as external reviewers to provide brief comments about the validity and usefulness of our methodology and results. Together with the study investigators, these external reviewers compared the list from round 3 with the new Swiss PROFILES, to assure consistency with the SWISS EPAs for undergraduate medical students and the new list of BTM competencies.

### Data handling

A descriptive analysis of each questionnaire result was conducted. Data from the consecutive rounds were stored to fulfill the requirements of the Swiss Research Act on the Departmental research server LabKey (LabKey Software, Seattle, USA), which was accessible only to the investigators through a personalized passwords. We followed the Guidance on Conducting and Reporting Delphi Studies (CREDES) [23].

## Results

The data were collected between the 1 October 2018 and 28 February 2019. Round 1 took 30 days and enrolled 18 participants out of the 36 invited (response rate 50%). The group description and participation rate are given in Table 1. Round 2 took 10 days, and round 3 took 14 days. There were no drop-outs after enrollment.

**Table 1:** Participation rate during the first round of the Delphi method

Type of Stakeholder	Invited	Accepted	Participation rate (%)
BTM teachers	6	6	100
Certified emergency physicians	6	4	66
Final-year medical students	6	2	33
Pre-hospital doctors	6	5	83
Curriculum designers	6	1	16
External experts	6	0	0
Total	36	18	Mean: 50

### First round results

The participants listed 47 priorities, 28 knowledge items, 30 skills, and 25 attitudes. These were organized into a framework that included nine domains of BTM, with the aim to compile the questionnaire for round 2; this ending up with 85 items.

These items were coded according to the following competencies: “triage” (7.4% of answers); “structured approach to trauma” (9.4%); “general trauma management” (15.3%); “technical skills” (23.5%); “particular trauma management” (15.3%); “transport” (3.5%); “human factors” (22.4%); “security issues” (4.7%): and “knowledge” (1.2% representation of all answers).

### Second round results

The second round response rate was 100%. The median and percentage of agreement for each item is shown in Table 2. Items with > 75% agreement and a median of 5 were accepted as consensual and did not enter round 3. Items with a median ≤ 3 and overlapping with subjects of other third-year courses were excluded.

**Table 2:** Competencies from Delphi round 2

Coding	Item	Median (IQR)	Agreement (%)	Result
Triage	Know how to conduct preclinical triage	3.5 (2.75–4)	50.0	
	Know how to conduct In-hospital triage	4 (2–4)	61.1	
	Demonstration of triage in a workshop	4 (2.75–4.25)	61.1	
	Do triage	**3** (2.75–4)	**38.9**	^b^Excluded
Structured approach	Describe the ABCDE algorithm to assess a trauma patient	**5**	**100**	Consensus
	Perform a secondary survey	4 (3–5)	72.2	
	Be able to describe the ABCDE sequence of trauma	**5** (4–5)	**100**	Consensus
	Do a primary survey according to the ABCDE approach	**5**	**100**	Consensus
	Have a structured approach to the patient	**5**	**100**	Consensus
	Treat only what needs to be treated, fast	4 (4–5)	88.8	
	Why the ABCDE is important: “To treat first what kills first”	**5**	**100**	Consensus
	Which problems to identify in primary and secondary survey	**5** (4–5)	**83.4**	Consensus
Management general	Diagnose and treat life-threatening conditions	**5** (3.75–5)	**77.8**	Consensus
	Treat hemorrhagic shock	**5** (4–5)	**88.9**	Consensus
	Teach/refresh the BLS	**5** (4–5)	**83.4**	Consensus
	Treatment of massive bleeding	4,5 (4–5)	88.9	
	Diagnostic tools in trauma management	3 (3–4)	44.4	^b^Excluded
	Trauma cinematics	3.5 (2,75–5)	50	^b^Excluded
	Be able to discuss the reason why ATLS is structured the way it is, with its benefits and pitfalls	4 (3–5)	72.2	
	Be able to stop the bleeding	5 (4–5)	**100**	Consensus
	What to do as first responder	4 (3–5)	72.2	
	Process of treatment of trauma patients from triage to definitive care	3 (2–4)	33.4	^b^Excluded
	The AMPLE	4 (3–5)	88.9	
	Use oxygen correctly	**5** (3.75–5)	**77.8**	Consensus
	Assessment of vital signs	**5** (4–5)	**100**	Consensus
Technical skill	Basic knowledge of immobilization techniques	**5** (4–5)	**88.9**	Consensus
	Indications, contra-indications, advantages and disadvantages of immobilization techniques	4 (3.75–4)	77.8	
	Immobilization skills	4 (3.75–5)	77.7	
	Demonstrate and use material for the management of trauma patients	4 (3–5)	72.2	
	Thorax drain insertion demonstration	3 (1.75–3.25)	22.2	^b^Excluded
	Pelvic sling use	**5** (3.75–5)	**77.8**	Consensus
	Airway management	4 (2.75–5)	61.1	^a^Excluded
	At the end of the course, students should be able to perform a simulated scenario	4 (4–5)	94.5	
	Perform bag-mask ventilation	4 (3.75–5)	77.7	
	Perform the airway management specific to trauma (MILS)	4 (2–4)	55.1	
	Perform HWS immobilization	5 (4–5)	**88.9**	Consensus
	Demonstrate the immobilization of a conscious trauma victim including extrication collar and pelvic binder	4 (4–5)	83.3	
	Demonstrate immobilization techniques and consequences for clinical work	4 (3–5)	72.2	
	Adequate handling of the spine, check that no danger threatens, since a paralysis is a problem		66.6	^c^Excluded
	Immobilization with extrication collar	4 (4–5)	94.4	
	Immobilization with vacuum mattress	4 (4–4.25)	83.3	
	Use of the scoop stretcher	4 (3–4)	61.1	
	Use of the spine board	4 (3–4)	72.3	
	Perform a log-roll	4 (4–5)	94.5	
	Perform an E-FAST	3 (1–4)	38.9	^b^Excluded
Management specific	Tourniquet use	4 (3–4.25)	66.6	
	Assessment of minor musculoskeletal trauma	3 (2–4)	27.8	^b^Excluded
	Assessment of traumatic brain injury	4 (3–5)	72.2	
	Wound care	3 (2–4)	44.4	^b^Excluded
	Aspects not included in ATLS, like coagulation aspects of trauma, use of painkillers	3 (2–4)	38.9	^b^Excluded
	Be able to discuss the pathophysiological changes in hypovolemic patients (why look at lactate and pH, not hemoglobin?).	4 (3–4.25)	66.6	
	Know the basics of diagnosis and treatment of different kinds of trauma (e.g., head, abdomen, thoracic, spinal, extremities)	4 (3–5)	66.7	
	To correlate trauma cinematics with potential lesions	4 (3–4)	61.1	
	Wound care	3 (2–4)	27.1	^b^Excluded
	Manage massive bleeding and perform the needle decompression of tension pneumothorax, because it saves lives	4 (2–5)	61.1	^c^Excluded
	Perform sutures	1.5 (1–3)	16.7	^b^Excluded
	Discuss the limitations of trauma care for the elderly	3 (2–4)	38.9	^b^Excluded
	Organization and reporting of the trauma scene	3 (2–4)	72.2	^b^Excluded
Transport	Transport to adequate hospital	4 (3–5)	61.1	
	Stay and play vs scoop and run	4 (3–5)	72.2	
	Safely transport a trauma patient	4 (2–4)	55.5	
Human factor	Teach non-technical skills (leadership, membership, situational awareness)	4 (3–5)	61.1	
	Teach about decision-making	4 (3–5)	66.6	
	Empowerment of students in the classroom	4 (3–4)	66.7	
	Teach about teamwork	4 (4–5)	83.4	
	Establish clear communication	4 (3.75–5)	77.7	
	Work as a team member	4 (3.75–5)	77.7	
	Be able to call for help properly	4 (3.75–5)	88.9	
	Coordination of different professional groups in an emergency	3 (2–3.5)	72.2	^b^Excluded
	Have communication skills	**5** (4–5)	**88.9**	Consensus
	Correctly communicate in a handover	4 (3–5)	66.6	
	Coordinate team members	3.5 (3–4)	50.0	
	Be able to lead a trauma situation	3 (2–3.25)	22.3	^b^Excluded
	Be a good team player	4.5 (4–5)	88.9	
	Learn to prioritize	4.5 (4–5)	94.0	
	Be aware of own limitations	**5** (4–5)	**100**	Consensus
	Be able to communicate clearly	**5** (4–5)	**94.4**	Consensus
	Be capable of decision-making	4 (3–4.25)	72.2	
	Stay calm	4 (3.75–5)	77.7	
	Speak up	4 (4–5)	83.3	
Security	Maintain safety of self, team, and patient	**5** (3.75–5)	**77.8**	Consensus
	Call for help properly and assess your security	**5** (4–5)	**94.5**	Consensus
	Secure the place and self	**5** (4–5)	**83.4**	Consensus
	Be able to assess your own security in the emergency location	**5** (4–5)	**88.9**	Consensus
Knowledge	The script of the BTM course Bern	4 (3–4.25)	72.2	^c^Excluded

^a^ Items with overlapping subjects with other current third-year courses

^b^Items with median ≤ 3

^c^Items excluded due to potential misunderstanding of phrasing

The overall agreement in round 2 was 87%. The overall agreement in “triage” was low (52%). No consensus was reached in 25% of the items, which resulted in their exclusion. “Structured approach” had high overall agreement (91%) and a consensus of 75%. “General management” had an overall agreement of 75% (high), with 23% of the items excluded and 46% consensus. “Technical skills” had moderate overall agreement (73%), with 10% item exclusion and 15% acceptance. “Specific management” showed low overall agreement (51%), with an item exclusion rate of 53%, and no consensus. “Transport” had low overall agreement (63%), and no item exclusion or agreement. “Human Factors” had high overall agreement (75%), with 11% of items excluded and 16% consensus. “Security” had 86% overall agreement and all of the items reached consensus. “Knowledge” excluded only one item due to misunderstandings in the phrasing. From the 85 items in round 2, only 44 showed disagreement and were taken up in round 3.

### Third-round results

All eighteen stakeholders assessed the 44 items for inclusion in the final curriculum (100% response rate). All of the competencies with an agreement of 75% or more were selected for the final listing. This round reached consensus for 20 items (45%). Overall agreement was 76%. “Triage”, “Structured Approach”, and “Transport” did not reach agreement. Higher agreement was reached for “General Management” (75%), “Human Factors” (64%), and “Technical Skills” (57%). Students should ***not*** be taught advanced airway management during the BTM course had 72% agreement, but they should be able to perform bag-mask ventilation correctly when deemed necessary (66.7% agreement). Table 3 shows the list of all of the included items. After merging the redundant items, we ended up with a list of 29 items to be included in the BTM course (Table 4).

**Table 3:** Items included by consensus from rounds 2 and 3 of the Delphi method and the subsequent merging and editing of competencies

Coding	Item	Action
Structured Approach (SA)		
SA 1	Describe the ABCDE algorithm to assess a trauma patient	Accepted in the final listing
SA 2	Be able to describe the ABCDE sequence of trauma	Same as SA 1 - Deleted
SA 3	Do a primary survey according to the ABCDE approach	Same as SA 1 - Deleted
SA 4	Have a structured approach to the patient	Same as SA 1 - Deleted
SA 5	Why the ABCDE is important: “To treat first what kills first”	Accepted in the final listing
SA 6	Which problems to identify in primary and secondary survey	Accepted in the final listing
Management General (MG)		
MG 1	Diagnose and treat life-threatening conditions	Accepted in the final listing
MG 2	Treat hemorrhagic shock	Merged with MG 4 and MG 5
MG 3	Teach/ refresh the BLS	Accepted in the final listing
MG 4	Treatment of massive bleeding	Merged with MG 2 and MG 5
MG 5	Be able to stop the bleeding	Merged with MG 2 and MG 4
MG 6	What to do as first responder	Same as MG 1 - Deleted
MG 7	The AMPLE	Accepted in the final listing
MG 8	Use oxygen correctly	Accepted in the final listing
MG 9	Assessment of vital signs	Accepted in the final listing
Technical Skills (TS)		
TS 1	Basic knowledge of immobilization techniques	Accepted in the final listing
TS 2	Indications, contra-indications, advantages and disadvantages of immobilization techniques	Accepted in the final listing
TS 3	Immobilization skills	Same as HS 1 - Deleted
TS 4	Demonstrate and use material for the management of trauma patients	Accepted in the final listing
TS 5	Pelvic sling use	Accepted in the final listing
TS 6	At the end of the course, students should be able to perform a simulated scenario	Accepted in the final listing
TS 7	Perform HWS immobilization	Accepted in the final listing
TS 8	Demonstrate the immobilization of a conscious trauma victim including extrication collar and pelvic binder	Accepted in the final listing
TS 9	Immobilization with extrication collar	Same as HS 8 - Deleted
TS 10	Immobilization with vacuum mattress	Accepted in the final listing
TS 11	Perform a log-roll	Accepted in the final listing
Management Specific (MS)		
MS 1	Tourniquet use	Accepted in the final listing
Human Factors (HF)		
HF 1	Teach non-technical skills (leadership, membership, situational awareness)	Accepted in the final listing
HF 2	Teach about teamwork	Accepted in the final listing
HF 3	Establish clear communication	Accepted in the final listing
HF 4	Work as team member	Accepted in the final listing
HF 5	Be able to call for help properly	Accepted in the final listing
HF 6	Have communication skills	Same as SS 3 - Deleted
HF 7	Correctly communicate in a handover	Accepted in the final listing
HF 8	Be a good team player	Merged with SS 4
HF 9	Be aware of own limitations	Accepted in the final listing
HF 10	Be able to communicate clearly	Same as SS 3 - Deleted
HF 11	Stay calm	Accepted in the final listing
HF 12	Speak up	Accepted in the final listing
Security (Sec)		
Sec 1	Maintain safety of self, team, and patient	Accepted in the final listing
Sec 2	Call for help properly and assess your security	Same as SS 5 and Sec 1 - Deleted
Sec 3	Secure the place and self	Same as Sec 1 - Deleted
Sec 4	Be able to assess your own security in the emergency location	Same as Sec 1 - Deleted

**Table 4:** Final items to include in the BTM course for third-year medical students of the University of Bern

Coding	Item
SA 1	Describe the ABCDE algorithm to assess a trauma patient
SA 5	Why the ABCDE is important: “To treat first what kills first”
SA 6	Which problems to identify in primary and secondary survey
MG 1	Diagnose and treat life-threatening conditions
MG 2	Treatment approaches to massive bleeding and hemorrhagic shock
MG 3	Teach/ refresh BLS
MG 7	The AMPLE (Allergies, Medication, Past history, Last eaten, Events)
MG 8	Use oxygen correctly
MG 9	Assessment of vital signs
HS 1	Basic knowledge of immobilization techniques
HS 2	Indications, contra-indications, advantages and disadvantages of immobilization techniques
TS 4	Demonstration and use of material for the management of trauma patients
TS 5	Pelvic binder use
TS 6	At the end of the course, students should be able to perform a simulated scenario
TS 7	Perform cervical immobilization
TS 8	Demonstrate the immobilization of a conscious trauma victim including extrication collar and pelvic binder
TS 10	Immobilization with vacuum mattress
TS 11	Perform a log-roll
MS 1	Tourniquet use
HF 1	Apply non-technical skills (leadership, membership, situational awareness)
HF 2	Teach about teamwork
HF 3	Establish clear communication
HF 4	Work as a team member and be a good team player
HF 5	Be able to call for help properly
HF 7	Correctly communicate in a handover
HF 9	Be aware of own limitations
HF 11	Stay calm
HF 12	Speak up
Sec 1	Maintain safety of oneself, team and patient

### External reviewing

External reviewers made comments on different aspects of the project: validity, applicability, usefulness of results, and adequacy of methodology for curriculum development. All of the experts mentioned the adequacy of panel selection for enhancing face validity. Reviewers also pointed out that the methodology involved was adequate to inform on competencies and curriculum development. However, our results were only considered applicable to the local standard of practice, because of the low response rate of the external sources.

The reviewers commented that our findings were useful because the mapping against the PROFILES report included a high percentage of items. Comparing the final list against the EPAs in PROFILES revealed agreement in 82% of all items of the new BTM course.

## Discussion

This study determined which core competencies are necessary to teach to third-year medical students in BTM based on a stakeholder needs assessment and the requirements of the new Swiss CBME curriculum PROFILES. Our three-round Delphi process involved all course stakeholders and included external reviewers for validation. Twenty-nine competencies were selected out of an initial 130, for the new teaching program for BTM in Bern.

In line with Greenhalgh (2014), we needed an alternative view on evidence-based medicine that emphasizes the value of expert judgement and that is not directly accessible through clinical trials. Our Delphi process allowed all of the rounds to be performed electronically. This cut costs, time, and resources [24]. Additionally, opinions could be expressed anonymously, to avoid peer pressure, as well as promoting new perspectives on the subject.

Our approach was especially helpful because the stakeholders came from different backgrounds and Departments. Performing face-to-face discussions would be very hard to organize, and would be impractical. All in all, the Delphi method was a quick way to achieve solid results. The most important competencies surfaced first, and remained after several rounds of reflection. Less important or not so clearly formulated competencies were systematically excluded. These advantages might explain the extensive use of the Delphi technique in medical education curriculum development [25–29].

### Limitations

We faced the usual limitations of the Delphi technique [20]. Participant commitment was substantial, as they needed to complete all three rounds. Our open questions might have discourage stakeholders from answering, and long questionnaires can decrease overall motivation to participate. All this might account for the 50% drop-out rate from the first to the second round of questionnaires. Additionally, there is no clear definition in the literature of what makes an “expert”. Nonetheless, our stakeholders were representatives of the groups that are directly related to BTM education at Bern University. By agreeing to participate, they showed a significant level of interest in the topic. Our panel consisted of 18 members, a number considered to be adequate to a Delphi method [20, 24]. The high response rates after enrollment also increased the validity of the results. Furthermore, the final list of competencies was validated by external reviewers with expertise in trauma medicine, which strengthens our findings.

The Delphi method is considered an effective tool to find “consensus”, although the level at which this “consensus” occurs is difficult to determine. The reported levels of consensus range from 51 to 80% [30], with a trend to higher percentages of agreement [23]. We set, a priori*,* a median score of 5 and > 75% agreement to accept a statement as consensual. Obviously, the sole determination of a consensus threshold does not mean the “correct” solution has been found [27]. Additionally, the Delphi technique tends to eliminate extreme positions and to force a conservative status [20].

Another limitation is reliability [31]. There is no evidence available to indicate whether two different panels given the same initial information will produce the same results. Therefore, generalizability might be limited by unique stakeholder characteristics, and solutions reached by such Delphi processes are simply a consensus opinion of this group.

The strengths of our study include the following: our approach was simple, easy, and effective in developing curricular adaptation. In this sense, this approach might be applicable to other curricula development, as it allows priorities for a mandatory clinical skills course for undergraduate medical students to be summarized in a short time and with limited resources. The involvement of all stakeholders and the fast turn-around of the three Delphi rounds assured integration of the current needs of teachers, students, and in- and out-of-hospital emergency physicians. The correlation to the legally given new national curriculum for the study of medicine addressed the needs, and fulfilled the responsibility for curriculum realignment.

The PROFILES report was, in our case, inadequate to effectively educate third-year, trauma-naïve, medical students, because PROFILES lists all of the competencies medical students need to have at the *end* of their training. Adapting this framework for the third-year course was challenging, and this was difficult to “fine tune”. However, the Delphi technique was particularly useful for the adaptation of BTM knowledge, skills and competencies for third-year medical students. Such an adaptation of competencies to a specific student level was evident in round 2 of this study, where the category “Specific Management” had low overall agreement on a variety of skills (50.7%), with 53% item exclusion and no consensus. In round 3, only the management of a tourniquet found agreement. Therefore, we could adapt the given trauma competencies to the third-year level. Our results determine which BTM principles third-year students should be exposed to. This has been done before in emergency medicine curricula [32–35], but our study uses the Delphi technique for the first time in a BTM curriculum.

We were surprised by the strong vote on the human factor competencies, which had not been addressed before in the BTM course. Our findings represent the expressed need to introduce teaching of non-technical skills beyond the “medical expert” competence. Human factors include a set of social and cognitive abilities that encompass situational awareness, risk assessment, clinical decision making, leadership, communication skills, and teamwork [36]. The influence of these human factors on clinical outcomes has already been ascertained [37, 38]. In the undergraduate setting, however, there is a substantial lack of guidance and teaching for these skills [39, 40]. Our stakeholders underlined the need to teach human factors, which might represent a trend that is already occurring in the postgraduate medical education *milieu*, as a shift towards a more holistic model of medical education.

## Conclusion

The revised BTM course curriculum proposed in this study is an attempt to tailor concepts and skills to fulfill unmet needs. It is an example of curricular adaptation driven by a new regulatory framework, to reform learning outcomes. In an effort to achieve this, a three-step Delphi process that involved all stakeholders of the course finally listed 29 core competencies to be taught to third-year medical students in the BTM course.

### Practice points

- Example of a curricular adaptation based on stakeholders’ needs assessment driven by a new regulatory framework.

## Data Availability

The datasets analyzed during the current study are available from the corresponding author on reasonable request.

## Abbreviations

BTM: Basic Trauma Management
CBME: Competency-based medical education
EPA: Entrustable professional activity
IQR: Interquartile range
PROFILES: Principal Relevant Objectives and Framework for Integrated Learning and Education in Switzerland
SLCO: Swiss Catalogue of Learning Objectives for Undergraduate Medical Training
